# From data-driven cities to data-driven tumors: dynamic digital twins for adaptive oncology

**DOI:** 10.3389/frai.2025.1624877

**Published:** 2025-07-25

**Authors:** Irem Karaman, Burhan Sebin

**Affiliations:** ^1^Sylvester Comprehensive Cancer Center, University of Miami Miller School of Medicine, Miami, FL, United States; ^2^Atlas Space, Miami, FL, United States

**Keywords:** digital twin, precision oncology, smart cities, multimodal data integration, cancer care

## Introduction

Oncology is undergoing a transformation due to the advent of digital twin technology, which enables precision therapy by creating synchronized virtual copies of physical systems. Unlike static models, dynamic digital twins continually integrate multimodal patient data such as clinical, imaging, and molecular data, to simulate therapy scenarios and direct real-time therapeutic decisions (Tortora et al., 2025). The success of smart city digital twins, which control complicated systems like traffic and energy in real time, provides a model for oncology (Peldon et al., 2024). Like cities, tumors are dynamic, multiscale systems shaped by therapy, immunological responses, and genetic alterations. There must be less trial-and-error in cancer care if we can use dynamic, learning-based twins to practice therapy instead of static ones that rely on pre-treatment snapshots, which miss this progression.

## The limits of static oncology twins

Static oncology twins are often created from a single pre-treatment dataset, which includes imaging and genetic profiles, and used to predict first medication responses. However, when tumors adapt into their environment and therapy, these one-time models lose accuracy, failing to predict emergent resistance mutations and microenvironmental modifications (Wang et al., 2024). Static twins are unable to detect early signals of relapse or toxicity because they exclude longitudinal indicators such as ctDNA kinetics, biomarker signatures, and routine labs, as well as real-time physiological data from wearables (Aghamiri and Amin, 2025). Moreover, while assays like Oncotype DX and spatial cell-type mapping inform initial risk stratification, they remain disconnected from iterative clinical decision loops (Chiru and Vetter, 2023). Integrative-cluster trials categorize patients based on a combination of molecular and histopathologic characteristics; however, they still do not incorporate closed-loop adaptation. On the other hand, a dynamic twin would actively adapt therapy by recalibrating its AI and mechanical models with each new data stream. Nonetheless, prospective validation via observational cohorts and randomized studies is necessary to establish the clinical utility of these adaptive systems.

## Smart cities as operational templates

**Figure 1:**
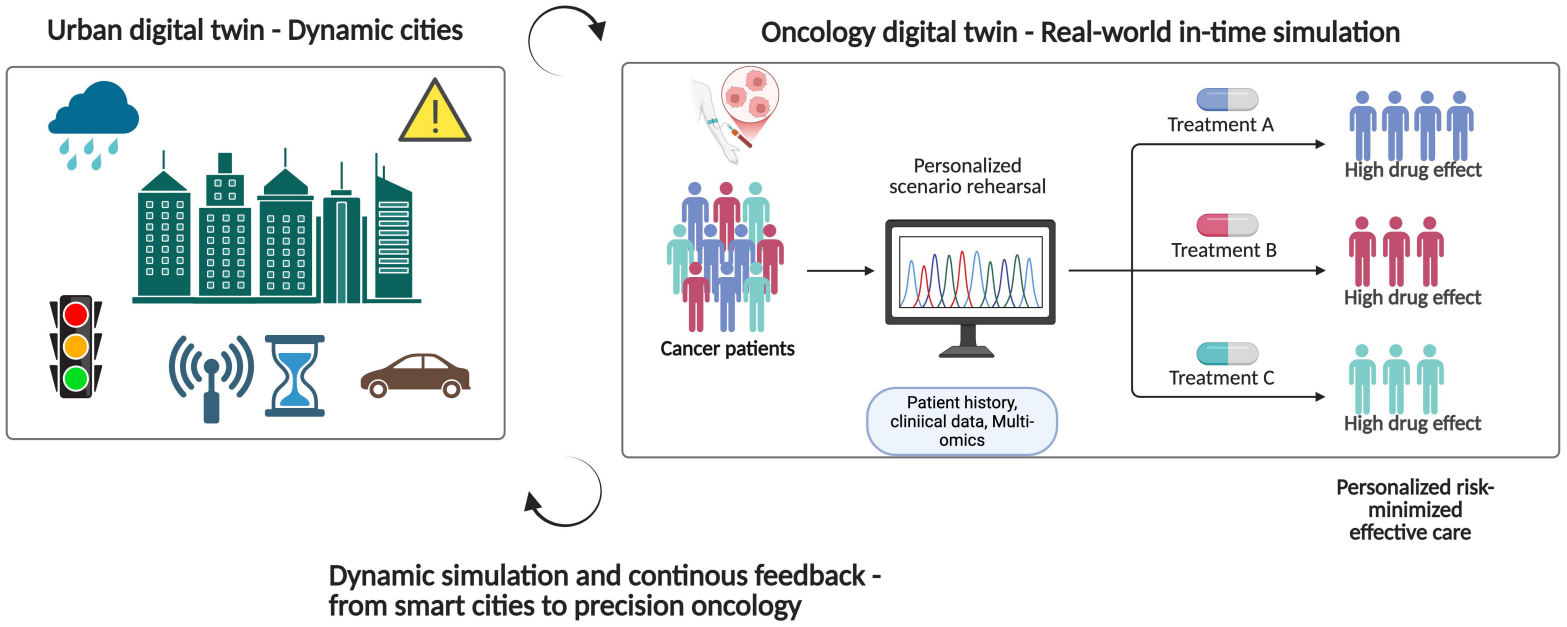
The goal of an urban digital twin is to simulate optimal city operations by ingesting real-time sensor data; a cancer digital twin, on the other hand, would combine genetic, imaging, and biomarker streams to practice treatments and provide risk-minimizing, individualized cancer care. Figure created with BioRender.

Digital twins in smart cities serve as central command centers for urban ecosystems, consistently integrating data from traffic cameras, smart streetlights, water distribution monitors, public transit GPS, and air quality sensors (Alvi et al., 2025). They function as a comprehensive citywide system, executing real-time simulations to evaluate the impacts of diverse scenarios, including the closure of a highway during peak hours, the reconfiguration of traffic signals to mitigate congestion, or the redirection of emergency response vehicles in unusual weather conditions. The results are then utilized to provide optimized control signals to the physical infrastructure (Huang et al., 2025). For example, *Virtual Singapore* uses real-time environmental and transportation data from over 30 agencies to simulate urban planning, emergency response, and energy efficiency, making it a global benchmark for centralized scenario rehearsal (Ferro-Escobar et al., 2022). The open digital twin of Helsinki facilitates public participation and climate planning via accessible 3D simulations, aiding in the visualization of solar potential and guiding zoning choices (Hämäläinen, 2021). Simultaneously, Shanghai's urban management twin enhances everyday operations and emergency response by combining IoT, AI, and real-time monitoring across districts, resulting in up to 30% gains in municipal efficiency (Wang et al., 2024a). Upon the activation of a flood alert, the twin can promptly simulate reservoir releases, road closures, and diversion routes, analogous to how an ICU dashboard predicts patient stability across various ventilator settings (Figure 1).

An oncology twin reflects this dynamic, feedback-oriented urban model. The system assimilates ongoing clinical notes and imaging data, analogous to how a city twin processes CCTV feeds (Wolf et al., 2022; Shen et al., 2025). It incorporates pathology reports, genomic sequencing, and biomarker trends, similar to the way environmental sensors monitor particulate levels. Heart rate, blood pressure, sleep patterns, stress levels, changes in glucose levels, and even gait analysis are all wearable streams that can be used like mobile noise or air quality tools to let the twin know about changes in the body (Nadeem et al., 2025). In order to allow predictive algorithms to make real-time adjustments to dosing regimens or therapy switches, the city's infrastructure is routinely audited using serial liquid biopsies and tissue samples. By considering the tumor and its host as an interdependent and dynamic system, similar to simulating rush-hour traffic and power-grid load, the twin may predict spikes in tumor growth, mutations that confer resistance, and areas of toxicity that are particularly harmful. This provides doctors with a real-time practice ground for therapy and individualized treatment plans.

## Case studies: dynamic twins in action

Recent efforts demonstrate the feasibility of dynamic oncology twins, with new use cases expanding their scope, summarized in Table 1.

**Table 1:** Representative dynamic twin case studies in oncology and smart city examples highlighting continuous data ingestion, scenario simulation, and adaptive feedback.

**Digital-twin application**	**Domain/ setting**	**Lead institution(s)**	**Core data and model**	**Reported outcome/insight**	**References**
Glioblastoma radiotherapy twin	Neuro-oncology	Oden Institute, UT Austin	Bayesian tumor-growth model updated with serial MRI during radiotherapy	Adaptive dosing delayed median progression by ≈6 days with lower total dose	Chaudhuri et al., 2023
LUNG-CANCER 3-D TWIN	Thoracic oncology	Stanford + NCI–DOE	Deep CNNs on CT, digital pathology, genomics; ctDNA updates	Reconstructs 3-D tumor, infers EGFR status, forecasts resistance, suggests therapy switches	Stahlberg et al., 2022
FarrSight virtual-trial twins	Multicancer trial optimization	Concr (VISION trial)	Patient-specific simulation of alternative regimens	Higher response when real therapy matched twin recommendation	Griffiths et al., 2024
Melanoma immunotherapy twin	Immuno-oncology	Indiana University	Multiscale agent-based immune–tumor model	Predicts immune escape; tests checkpoint sequencing	Shmulevich and Aguilar, 2022
Pain-management twin	Supportive care/PK-PD	Multi-center	Population PK/PD model of fentanyl	Optimizes dosing, reduces adverse events	Cukic et al., 2024
Personalized AML chemo scheduler	Hematology	Academic consortium	Longitudinal counts + mechanistic kinetics	Avoided leukopenia in 10/13 AML cases	Jost et al., 2020
Breath-gas early-detection twin	Non-invasive screening	Industry–academic group	Volatile metabolite ML signatures	Detects pre-clinical tumor shifts via breath	Chung et al., 2022
Clinical-trial design twin	Trial optimization	Multi-center	Virtual cohorts predicting toxicity vs efficacy	Reduced adverse events in trial simulations	Susilo et al., 2023
Smart-city traffic twin	Urban operations	City of Singapore—“Virtual Singapore”	Live traffic, IoT, weather feeds + agent-based model	Real-time rerouting cut congestion by ~15 %	Ferro-Escobar et al., 2022
Shanghai municipal digital twin	Urban operations	Shanghai Municipal Government; Smart City Program	City-wide IoT sensor network, AI analytics, real-time cross-district monitoring	Up to 30 % gain in municipal efficiency and faster emergency response	Wang et al., 2024a

These case studies highlight dynamic twins' core functionalities: real-time data assimilation, multiscale modeling, and therapy rehearsal, extending to education and trial design.

Digital twins in oncology must integrate patient data from diverse sources alongside the expertise of oncologists, clinical guidelines, and pertinent decision-making criteria. This ensures that the twin can demonstrate decision-making processes in a more intricate manner, particularly when it must evaluate individual critical criteria rather than solely relying on raw data streams. Incorporating domain-specific knowledge, such as the relative importance of prognostic signs or patient comorbidities, into digital twin recommendations ensures that they are consistent with established therapeutic rationale. Instead of adopting a one-size-fits-all approach, oncology digital twins should be created for each type of cancer since tumors evolve and treatments vary. For example, a glioblastoma twin must consider the tumor's dissemination and its responsiveness to radiation therapy, whereas a breast cancer twin would emphasize the functionality of hormone receptors and the malignancy's sensitivity to chemotherapy (Chaudhuri et al., 2023). Conversely, lung cancer twins consider mutational profiles such as EGFR or ALK status when determining targeted therapy options (Stahlberg et al., 2022). Numerous patient data exist within EHR/EMR systems; nevertheless, the generation of real-time digital twins is challenging due to data silos, inconsistent formatting, absent longitudinal records, and delays in data acquisition. Confronting these difficulties requires the synchronization of data streams, the establishment of interoperability standards, and the assurance of real-time data that is readily available. Contemporary clinical monitoring systems also have challenges in accurately modeling tumor development, medication resistance, and treatment-related toxicity at the individual level. Challenges involve the limited resolution of conventional imaging techniques, insufficient liquid biopsy collection, and inadequate real-time biomarker surveillance, including circulating tumor DNA or PD-L1 fluctuations (Susilo et al., 2023). More precise and dynamic depictions of disease progression and treatment effect are made possible by new technology that are rapidly improving, such as high-frequency wearable biosensors, serial liquid biopsy platforms, and advanced imaging methods.

Dynamic digital twins in cancer care, which evolve in response to evolving patient data, are distinct from static ones that only run once. FarrSight^®^-Twin perpetually integrates novel genetic variants, does repeated whole-slide scans, and incorporates time-stamped clinical events, thereby recalibrating its predictive model with each update to predict the responses of breast cancer patients to treatment and immunotherapy (Griffiths et al., 2024). The Stanford–NCI-DOE lung cancer twin integrates follow-up CT images, interval pathology samples, and novel genetic and clinical data through a systematic process. Each update recalibrates the tumor growth and treatment response trajectories, transforming the model from a static representation into a dynamic virtual patient (Stahlberg et al., 2022). During each MRI session—T1-contrast, T2-FLAIR, and diffusion—the Bayesian engine assimilates the new voxel-level contours and ADC measurements, updates the coefficients for each patient's proliferation, invasion, and radio sensitivity, and subsequently recalculates iso-dose maps and fractionation. This loop transforms the UT Austin glioblastoma twin into a dynamic therapeutic guide, capable of adjusting treatment intensity in response to emerging infiltrative areas or reducing dosage upon confirmation of tumor shrinkage (Chaudhuri et al., 2023). This enables physicians to formulate therapy protocols that are more efficacious in decelerating the disease and mitigating its damage.

## Technical foundations of dynamic oncology twins

The foundation of every interactive digital twin is a solid data flow. For oncology, this entails combining several patient data sets into a single repository, such as EHRs, lab findings, radiographs (CT, MRI, PET), histopathology reports, liquid biopsies (circulating tumor DNA), and multi-omics datasets (genomics, transcriptomics, proteomics). Complying with established standards is essential for achieving interoperability. These standards include the OMOP Common Data Model for observational health data and the HL7 Fast Healthcare Interchange (FHIR) for clinical and imaging metadata (Vorisek et al., 2021). Additionally, automated extraction workflows and streaming APIs guarantee that new patient measurements are transferred into the counterpart with minimal latency, thereby maintaining real-time fidelity to the changing disease state (Ianculescu et al., 2025). Upon establishment of these streams, Apache Kafka facilitates the real-time transfer of clinical notes, imaging files, and multi-omics results, encapsulated in FHIR, OMOP, DICOMweb, or Phenopackets formats (Iancu et al., 2024). The incoming messages are stored in an RDF database that associates each data point with terms from SNOMED CT, LOINC, OncoKB, and NCIt, followed by the application of a variational auto-encoder to address any gaps (Touré et al., 2023). Subsequently, modality-specific AI models operate with high efficiency: 3D UNet++ and DenseNet-121 for CT/MRI, a Swin-Transformer for whole-slide images, LoRA-tuned DNABERT-2 for genomic variants, a Temporal Fusion Transformer for irregular lab series, Hetero-GraphSAGE for knowledge graphs, physics-informed networks for tumor growth equations, and a PPO agent that weighs projected survival benefits against toxicity (Yang et al., 2025). Hybrid models that integrate physics-informed and data-driven approaches utilize multimodal embeddings, including cross-attention early fusion, Bayesian late fusion, and tensor-gated hybrid fusion. They disseminate calibrated uncertainty, enabling ~1,000 therapy-rehearsal simulations to conclude in under 1 s, accompanied by median projections and 95% prediction intervals (Kemkar et al., 2024).

A flexible modeling system that integrates data-driven AI with mechanistic simulations is equally essential. Convolutional neural networks and transformer models can derive predictive characteristics from imaging and genomic sequences, respectively, whereas systems of ordinary and partial differential equations represent tumor development dynamics and drug–tumor interactions (Wang et al., 2024b). Agent-based models mimic microenvironment dynamics and immune-cell infiltration; physics-informed neural networks apply biological limits on acquired representations (Raissi et al., 2019). Generative methods, like variational autoencoders and generative adversarial networks, let you do “what-if” studies by putting together virtual groups of people who would be treated differently.

The scenario simulation engines that are built on top of these models serve as platforms for the rehearsal of virtual therapy. The twin predicts important results including tumor shrinkage, resistance emergence, and toxicity profiles by listing potential treatment plans, which may include different medication combinations, dosage regimes, or sequence orders (Griffiths et al., 2024). Utilizing reinforcement-learning algorithms allows for the optimization of therapeutic methods in pursuit of multi-objective goals, such as maximizing progression-free survival while minimizing side effects. New patient data is constantly being used by these algorithms to update policy judgments. Lastly, simulation at the point of care must be scalable and have minimal latency, and this can only be achieved with a solid computing foundation. Cloud-native architectures facilitate flexible resource allocation, whereas high-performance computing clusters or GPU-accelerated environments enhance the training of large-scale mechanistic and AI models. Edge-AI deployments, when integrated within imaging devices or wearable monitors, have the capability to preprocess data locally, enabling rapid interpretation and subsequently transmitting distilled features back to the central twin (Xu et al., 2025). The integration of these technical foundations establishes a robust and adaptable platform for the dynamic rehearsal of therapy digital twins within the field of precision oncology.

## Clinical validation and implementation

These projects possess significant potential; yet, they also present specific challenges that hinder their application in clinical environments. For instance, there is a lack of comprehensive real-time data integration, insufficient validation in prospective clinical trials, and a lack of clarity regarding the functionality of the models. To achieve FDA-approved digital twins, it is essential to ensure that clinical decision-making is safe, effective, and reliable through comprehensive multi-center validations, precise uncertainty quantification, transparent reporting, and integration into regulatory frameworks. Successful clinical use of digital twins requires their seamless integration of continuous multimodal data, quick and transparent prediction, assurance of privacy compliance, thorough validation, and support of clinical decision-making.

To put dynamic oncology twins into practice, a phased validation strategy is needed. This strategy entails retrospective *in silico* trials, which involve replaying historical treatment data to measure predictability, and prospective observational studies, which compare real-time twin predictions with actual patient outcomes, such as progression-free survival and response rate. Eventually, randomized trials will be required to determine whether twin-guided therapy enhances results compared to standard care, with early glioblastoma and immunotherapy pilots showing both potential and physician acceptance (Chaudhuri et al., 2023). The integration of visual outcome forecasts and explicit uncertainty estimates into EHR interfaces or tumor boards improves transparency and trust without increasing the workload. Scalable, multi-institutional implementation will necessitate secure, privacy-preserving designs like federated learning, common APIs, and ethical supervision.

## Governance, ethics, and future challenges

Protecting patients' private health information and other sensitive data from unauthorized parties is of the utmost importance. Federated learning techniques enable model training across universities without the exchange of raw data. This preserves confidentiality while facilitating collaborative learning. End-to-end encryption, comprehensive audit trails, and differential privacy protocols are further precautions that mitigate the risk of re-identification and ensure the model's accuracy (Khaled et al., 2025). Comprehending the process of prediction and allowing physicians to evaluate its reliability remains a significant challenge. To clarify this, it is necessary to incorporate explainability tools such as Shapley values or attention visualizations in complex hybrid models. A shift away from static decision-support aids and toward adaptive, continuously learning systems would necessitate new regulatory frameworks. These frameworks must delineate the distinction between recommendations and definitive clinical determinations. Formal regulations must ensure that developers, physicians, and institutions collectively assume responsibility for collaboratively developed guidelines in an equitable manner. Training datasets should include diverse patient demographics and bias auditing at each phase with clear reporting to improve fairness. Operational challenges, including increased processing demands and the integration of twin-generated insights into clinical workflows, can be minimized by employing surrogate modeling techniques, optimizing coding practices, and providing specialized training for diverse teams to understand and apply these insights (Zhang et al., 2025; Hasanzadeh et al., 2025).

## Future directions

By moving beyond individual therapy rehearsal, dynamic oncology twins have the potential to revolutionize precision medicine, speed up biomarker discovery, and provide insights at the population level. Collecting anonymized digital twin trajectories from a large number of patients may help researchers find new predictive biomarkers and treatment-response patterns that are not evident in single-cohort studies. Continuously adding wearable sensor streams that record physiological signals, exercise measures assure to improve twin fidelity, making it possible to watch and intervene in outpatient settings in real time. Where conventional healthcare infrastructure is lacking, lightweight twin platforms accessible through mobile devices and driven by reduced-order models have the potential to revolutionize cancer treatment in underserved areas. The foundation for large-scale clinical use of dynamic twins will be established by large-scale partnerships like the NCI-DOE Cancer Patient Digital Twins for Predictive Oncology program, which intends to specify shared validation standards, interoperable infrastructures, and regulatory procedures.

## Conclusion

Tumor twins, like smart cities, has to move away from static, one-time models and toward continually learning twins in order to improve urban infrastructure. These adaptive twins may leverage cloud-native and edge-AI computing platforms to integrate multiscale tumor models with streaming clinical, imaging, and genetic data. This facilitates physicians to dynamically tailor treatment by simulating therapy regimens *in silico*. The revolutionary potential of oncological twins is demonstrated by early case studies in areas such as adaptive immunotherapy dose, minimal residual disease monitoring, and chemotherapy scheduling. In the meantime, as high-performance infrastructures and data standards become widely available, validated therapy-rehearsal twins will be able to act as clinical copilots, keeping up with the changes in tumors and combining the engineering accuracy of smart cities with AI and precision medicine improvements. A new age of adaptive, patient-centric cancer treatment is emerging, and the intersection of these fields is the clear way forward.
